# Double Stranded RNA in Human Seminal Plasma

**DOI:** 10.3389/fgene.2017.00154

**Published:** 2017-10-18

**Authors:** Maxim V. Zagoskin, Richard E. Davis, Dmitry V. Mukha

**Affiliations:** ^1^Laboratory of Genetic Basis of Biodiversity, Vavilov Institute of General Genetics, Russian Academy of Sciences, Moscow, Russia; ^2^Department of Biochemistry and Molecular Genetics, University of Colorado School of Medicine, Aurora, CO, United States

**Keywords:** double stranded RNA, human seminal plasma, phenotype of offspring, telegony, genetic molecular markers

## Abstract

Recently, human semen was shown to contain cell-free nucleic acids, such as DNA, long single stranded RNA, and small RNAs–miRNA and piRNA. The RNAs have been suggested to have potential biological roles as communication molecules between cells and in the temporal and spatial regulation of gene expression in the male reproductive system. Here we demonstrate that human seminal plasma contains a variety of cell-free dsRNAs, describe a robust method to isolate this type of nucleic acid in preparative amounts, and discuss the potential biological roles of these molecules in inheritance. dsRNA plays a role in a variety of biological processes, including gene regulation, is extremely stable and can gain access to cells from the extracellular medium. We suggest that one of the possible functions of dsRNA in human seminal plasma may be to influence human oocytes and therefore, influence the offspring. It also remains possible that these dsRNAs might have potential use as biomarkers for the study of human physiopathological conditions and genetic variation.

Semen, also called seminal liquid, is a fluid that is emitted from the male reproductive tract and contains sperm cells capable of fertilizing the eggs of the female. The major component of semen is seminal plasma, which helps to maintain the sperm cell viability. Sperm cells make up only a small portion of the whole semen, from 1 to 5% of the total volume (Owen and Katz, 2005).

The seminal plasma is a mixture of components produced by several glands. These components are incompletely mixed during ejaculation; hence, the initial ejaculate is not an entirely homogeneous mixture. The chemical composition of human seminal plasma has been the subject of many studies. Recently, cell-free nucleic acids, such as DNA, long single stranded RNA, and small RNAs–miRNA and piRNA, were identified in human seminal plasma (Huang et al., 2009; Li et al., 2009, 2012; Hu et al., 2014). The biological role of cell-free seminal DNA and RNA remains unknown. One possible function of the cell-free RNAs in seminal plasma may be to contribute regulatory information between cells (possibly including sperm cells) and contribute to changes in gene expression. The RNA may provide key temporal and spatial regulation of gene expression in the testis and epididymis required for normal spermatogenesis and sperm maturation (Li et al., 2012).

Nucleic acids in human seminal plasma may also influence human oocytes. Genetic material present in seminal plasma might contribute to phenotypic changes in the developing zygote, and therefore, influence the offspring. This would constitute an additional level of gene regulation and hereditary information and represents an intriguing idea. Nucleic acids that are stable for a relatively long time, are resistant to the relatively aggressive environment of the female reproductive tract, and can penetrate into cells from the extracellular medium could contribute to novel mechanisms of inheritance. Moreover, penetration of these molecules into the precursors of mature oocytes, i.e., into the cells of the female germ line located in the ovary, could allow offspring to inherit the characteristics of a previous mating of the female parent (telegony) (Crean et al., 2014).

A likely candidate for the role of such molecules is double stranded RNA (dsRNA). dsRNA is involved in numerous biological processes and at least three different pathways can respond to dsRNA in mammals, including sequence-independent interferon response, editing by adenosine deaminases, and sequence-specific RNA interference (RNAi) (Chalupnikova et al., 2013). Mechanisms of RNAi, including sequence-specific degradation of RNAs complementary to the sequences of the dsRNA, translation inhibition, and RNAi-mediated methylation of genomic DNA altering the pattern of gene activities would likely be strong candidates for regulatory molecules in seminal fluid (for reviews, see Carthew and Sontheimer, 2009; Ghildiyal and Zamore, 2009). These RNAi mechanisms can contribute to epigenetic inheritance influencing the phenotype of future offspring (Waldron, 2016). Notably, RNAi pathways are known to be active, for example, in mouse oocytes (Wianny and Zernicka-Goetz, 2000; Grabarek et al., 2002; Svoboda, 2004; Nejepinska et al., 2012), in human oocytes (Homer et al., 2005), and in other human cells (Kawasaki and Taira, 2004; Gantier and Williams, 2007). dsRNA is extremely stable and is known to regulate gene expression in nematodes and insects and has been introduced through feeding of dsRNA or the microinjection of dsRNA solution under the cuticle (Kaletta and Hengartner, 2006; Huvenne and Smagghe, 2010). Semen dsRNA could be stable in the female reproductive tract for a relatively long time and gain access to the female germ cells and/or precursors thereby influencing the phenotype of future offspring. However, to date it is not known whether dsRNA is present in seminal fluid and whether it can gain entry into oocytes. Studies on the effect of dsRNA molecules on mammalian oocytes, including human oocytes, have been carried out using *in vitro* experiments based on microinjection or electroporation techniques. However, several lines of evidence from a variety of studies suggest that dsRNA can gain entry into cells (for reviews, see Bumcrot et al., 2006; Czech et al., 2011).

Little has been done to identify human seminal plasma cell-free dsRNA. In this paper, we describe a robust method to isolate this type of nucleic acid in preparative amounts and demonstrate that human seminal plasma contains a variety of extracellular dsRNAs. The described method will facilitate the analysis of the biological role of these molecules. Moreover, future comparative deep sequencing of seminal plasma dsRNA of different individuals will enable searching for new potentially informative genetic markers that could reveal the difference between human male individuals due to differences in the patterns of genome transcription and dsRNA formation in tissues related to seminal plasma production.

Semen samples were obtained by masturbation from three 30-year-old healthy men. All donors were fertile according to the data given in the sperm donor questionnaire. Donors abstained from sexual activity for >72 h before semen donation. Complete ejaculate samples were collected in sterile containers. This study was reviewed and approved by the Ethics committee of Vavilov Institute of General Genetics, Russian Academy of Sciences. All subjects gave written informed consent in accordance with the Declaration of Helsinki.

The extracellular dsRNA was enriched and purified from the semen using fractionation by equilibrium centrifugation in a CsCl-ethidium bromide density gradient. A similar approach has been previously used in our laboratory for the purification of dsRNA of a Drosophila reovirus-like virus with a segmented genome (Pasyukova and Mukha, 2009).

To remove the cells and cell debris from the semen, we used two different approaches. In the first approach, CsCl was added to the freshly collected semen to give a saturated salt solution. Under these conditions, proteins were denatured and an amorphous mass was generated that contains all cellular material. Centrifugation at 4°C and 16,000 g for 10 min was performed to remove the cells and cellular debris. The supernatant was carefully collected for subsequent assays. In the second approach, the freshly collected semen was twice centrifuged at 4°C at 1,600 g for 10 min and then 16,000 g for 10 min, respectively, to remove cells and cell debris. The supernatant (seminal plasma) was carefully collected, and CsCl was added to saturation. The nucleic acids isolated appear identical from the two methods.

Samples saturated with CsCl were diluted to a refractive index 1.3865 and ethidium bromide added to 0.8 mg/ml, similar to method used for purification of closed circular DNA (Sambrook et al., 1989), and were centrifuged at 45,000 rpm for 48 h (Beckman Spinco L2-65B, Ti50 rotor). After centrifugation, two fractions corresponding to linear DNA generating a band in the middle of the tube) and a pellet (on the bottom of the tube) were observed under UV light (365 nanometers). A broad diffuse fraction of nucleic acid was also observed between the banding DNA and pellet. A schematic representation of the detected fractions is shown in Figure 1A. The diffuse fraction (“Analysed fraction” in Figure 1A) was collected, CsCl and ethidium bromide were removed using standard protocols, nucleic acids were ethanol precipitated, and dissolved in nuclease free water.

**Figure 1:**
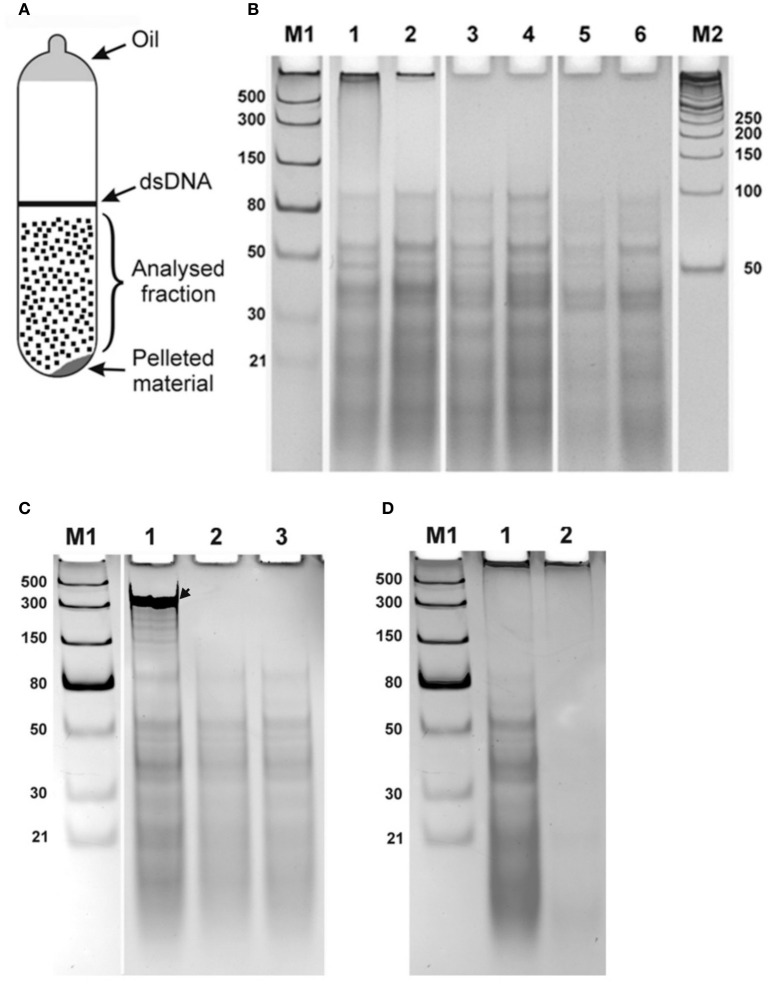
Double stranded RNA from human seminal plasma. **(A)** Schematic representation of UV-visible fractions after equilibrium centrifugation of the human seminal plasma from CsCl-ethidium bromide density gradients. **(B)** Electrophoretic separation on a 10% polyacrylamide gel of the isolated diffused fraction of nucleic acids collected after centrifugation (“Analysed fraction” in **A**). Nucleic acids isolated from seminal plasma of two different individuals are shown in lanes with even and odd numbers, respectively. 1, 2—Isolated material; 3, 4—Material treated with DNase I; 5, 6—Material treated with DNase I and RNase One Ribonuclease. The fraction located near the top of the gel was digested with DNase I treatment. However, the distinct bands spread over the region from ~100 bp to the bottom of the gel were resistant to both enzymatic treatments. **(C)** Enzymatic treatments of CsCl dsRNA fractions. Each sample was spiked with an *in vitro* synthesized ssRNA, see arrow, as an internal control, treated with different nucleases, and then separated by electrophoresis and visualized. 1, DNase I treatment. 2, RNase One Ribonuclease treatment. 3, RNase R treatment. Both RNase One and RNase R ribonucleases digest the spiked in ssRNA but do not act on the nucleic acid, dsRNA, from the seminal fluid. **(D)** Electrophoretic separation of seminal plasma nucleic acids 1 without and 2 after treatment with RiboShredder RNase Blend. All RNAs in the sample were completely degraded. M1 and M2—ladders (dsRNA Ladder and 50 bp DNA Ladder, respectively).

Electrophoretic separation of semen nucleic acid from different donors on a 10% polyacrylamide gel looked the same among the samples (Figure 1B illustrates this for two different donors, lanes 1–2). Nucleic acids observed included material near the top of the gel >1,000 bases, and more than a dozen distinct bands over a background smear ranging from ~100 bp and smaller.

We next analyzed the nuclease sensitivity of these nucleic acids using DNase I (Thermo Fisher Scientific, USA) and RNase One Ribonuclease (Promega, USA) according to the manufacturers' instructions to determine the nature of the nucleic acids. Electrophoretic separation of the semen plasma nucleic acids after DNase I treatment is shown in Figure 1B, lanes 3 and 4 (two different donors), and after treatment by both DNase I and RNase One Ribonuclease in Figure 1B, lanes 5 and 6 (two different donors). The fraction located near the top of the gel (Figure 1B, lanes 1 and 2) was digested with DNase I treatment, indicating that this fraction is represented by DNA molecules. However, the distinct bands spread over the region from ~100 bp to the bottom of the gel were resistant to both enzymatic treatments. These data suggest that these molecules are not DNA or single-stranded RNA and might correspond to dsRNA molecules. To further evaluate the nature of the nucleic acid, a ssRNA (250 b) was synthesized using the Riboprobe *in vitro* Transcription System (Promega, USA), mixed with the material obtained after DNase I treatment of the semen plasma nucleic acids, and the mixture was treated with RNase One Ribonuclease (Promega, USA) and RNase R ribonuclease (Epicentre, USA). Both RNase One and RNase R ribonucleases digested the added ssRNA but did not act on the material isolated from the seminal plasma (Figure 1C). Overall, this further suggests that the seminal plasma material consists of dsRNA molecules with 3′ overhangs shorter than seven nucleotides as RNase One endoribonuclease digests all types of ssRNAs including circular RNAs and the RNase R exoribonuclease digests all linear RNAs except for double-stranded RNAs with 3′ overhangs shorter than seven nucleotides (http://www.epibio.com/enzymes/nucleases-glycosylases-dna-binding-proteins/rna-exonucleases/rnase-r?details). Finally, RNA from the seminal plasma was treated by RiboShredder RNase Blend (Epicentre, USA) (an RNase blend that degrades both ssRNA and dsRNA, http://www.epibio.com/docs/default-source/protocols/riboshredder-rnase-blend.pdf; http://www.epibio.com/docs/default-source/forum-archive/forum-08-1—riboshredder-rnase-blend-destroys-unwanted-rna-quickly-and-efficiently.pdf?sfvrsn=6) which resulted in the complete degradation of all RNAs in the sample (Figure 1D).

The origin of the dsRNAs in seminal fluid remains unclear. They may be formed as a result of digestion of native duplex RNA molecules by a cellular RNase and thus correspond to native RNA hairpins or derived from RNA duplexes derived from the annealing of ssRNA molecules corresponding to the transcripts of the “+” and “–” DNA strands of the genome. The smaller dsRNA bands ranging from 18 to 32 bp may represent miRNA and piRNA precursor duplex molecules. Some of the dsRNA molecules may represent partially degraded dsRNAs from one or few dsRNA species.

In summary, we have shown that human seminal plasma contains a repertoire of cell-free dsRNA. We do realize that the identity, source, and functioning of these dsRNA are yet undetermined. We hypothesize that these dsRNAs could influence the implementation of genetic information or gene regulation in offspring and, if future sequencing reveals polymorphisms in dsRNA nucleotide composition among individuals, characterization of seminal fluid dsRNAs might be potentially useful as non-invasive molecular markers.

To date, the effects of endogenously derived dsRNA contributing genetic information or regulating gene expression in somatic eukaryotic cells are not well-characterized. Furthermore, the role of these molecules in the process of fertilization and early embryonic development remain largely unexplored, particularly with respect to the role of dsRNA in the process of human fertilization and epigenetic regulation of the embryonic development. The known properties and functions of dsRNAs and their identification in seminal plasma now enables future testing of a number of hypotheses including:

- The ability of dsRNAs to penetrate into mammalian oocytes can be tested for example in experiments with fluorescently labeled dsRNAs purified from human seminal plasma.
- To explore potential differences in the dsRNA complexity in different human male seminal plasma, which may be the result of differences in the patterns of genome transcription and dsRNA formation in tissues related to seminal plasma production, sequencing of dsRNA samples obtained from different individuals can be carried out.
- To determine if dsRNA from a seminal plasma may contribute to phenotypic traits of the progeny, experiments with a mix of dsRNA and semen using different mammalian species with various phenotypes (for example, dogs) may be performed.

We believe that the initial description of dsRNAs in seminal plasma and its possible influences offer intriguing perspectives for further research.

## Author contributions

MVZ: The acquisition, analysis, and interpretation of data for the work, and revising the manuscript critically for important intellectual content. RED: Analysis of data for the work and revising the manuscript critically for important intellectual content. DVM: Design of the work, the acquisition, analysis, and interpretation of data for the work, drafting the manuscript and revising it critically for important intellectual content. All authors approved the version to be published and agreed to be accountable for all aspects of the work.

### Conflict of interest statement

The authors declare that the research was conducted in the absence of any commercial or financial relationships that could be construed as a potential conflict of interest.
